# Formulation of a thermo-sensitive hydro-gel for ulcerative colitis treatment

**DOI:** 10.6026/97320630018925

**Published:** 2022-10-31

**Authors:** Deepa Sathesh, K Sathesh Kumar, Velmurugan Devadasan, Sujatha Kuppusamy

**Affiliations:** 1 Sri Ramachandra Faculty of Pharmacy, Sri Ramachandra Institute of Higher Education and Research (DU), Porur, Chennai, India; 2 Office of Dean Sponsored Research, Publications and Collaboration and Director R & D Cell, AMET University, Kanathur, ECR road, Chennai, Tamil Nadu, India

**Keywords:** Collagen, in situ hydrogel, Fucoidan, Ulcerative colitis, Docking, Mesalamine

## Abstract

Ulcerative colitis (UC) is a type of inflammatory bowel disease (IBD) that causes chronic intestinal inflammation in gastrointestinal (GI) tract, mainly in innermost lining of colonic mucosa. In any of the UC drug therapy regimens, maintaining remission is challenging and about 20-40% of patients don't respond to conventional UC medications, namely, amino salicylates, steroids and immunosuppressive drugs. These agents can weaken the patient's immune system thus enhancing the risk of infectious diseases. Therefore, in our exploration we probed to test marine-derived anti-inflammatory compounds as potential agents to treat UC. Fucoidan, a complex fucose-rich sulphated polysaccharide originated in edible brown algae with known anti-inflammatory properties was isolated from Turbinaria ornate. Collagen (Achillis tendon) is another agent that may provide a beneficial effect in wound healing and tissue regeneration. Collagen was also reported to possess anti-UC properties. Collagen has a limitation of being in solution form even at high concentrations. We therefore formulated fucoidan with collagen that underwent a sol-gel transition and yielded a gel like consistency in situ. This formulation showed sustained release of fucoidan for about 12 hours. The fucoidan, collagen and the fucoidan-collagen formulation were tested in the dextran sodium sulfate (DSS) induced colitis model in mice. In comparison to the vehicle treated group, fucoidan-collagen hydrogel formulation led to significant reduction in the clinical scores and rectal bleeding, which was higher than the reference standard, mesalamine and those seen with fucoidan and collagen given alone.

## Background:

Ulcerative colitis is an inflammatory bowel disease that can significantly impact the quality of life in affected individuals [1]. Maintaining remission is not possible in the long term with glucocorticoids due to safety concerns [2]. Immunosuppressive agents may pose a risk of microbial infections to patients [3]. Certain anti-TNF-alpha monoclonal antibodies such as infliximab and adalimumab show limited efficacy (with about 20-40% of non-responding patients). Furthermore, their use may be limited due to serious adverse events such as infections and secondary tumours [4]. Vedolizumab (VDZ), a monoclonal antibody against α 4β 7 integrin, VDZ has been shown to be effective as induction and maintenance therapy in UC. The cost of therapy with biologics can be significantly high compared to small-molecule agents in current use [5, 6]. Overall, existing therapies do not ensure a long-term clinical remission in UC patients, and are often associated with adverse effects. The need for newer therapeutic options for treating UC is thus warranted. [7-9]. Fucoidan is a complex fucose-rich sulphated polysaccharide originating from edible brown algae and is well-known for having multiple bioactivities, including strong anti-inflammatory effects. Though all brown seaweeds are rich in fucoidan, based on the abundant availability and ability to provide a good yield of crude fucoidan T. ornate was selected for this study. However, as per the literature [10] the position of the sulphate group, sulphate content, L fucose and percentage of other constituents play a role in their pharmacological activity. The chemical composition of fucoidan isolated from T. ornate found to contain a high percentage of fucose, sulphate, free sugars, protein, uronic acid, and other components [11]. Henceforth, it was found to possess good or enhanced pharmacological activity, especially anti-inflammatory [10], anti-oxidant [12], immunostimulatory [13], antiulcer, wound healing properties [14], etc. Hence, Turbinaria species were selected for further studies of isolating fucoidan. However, as discussed before, the effectiveness of these biological properties is related to the structure and composition of fucoidan, which in turn depends upon the source and extraction method. There is only a small amount of experimental research on fucoidan related to its pharmacokinetics profiling called ADME. However, fucoidan may still be present with favourable pharmacokinetics in relation to toxicity; the information on its biodistribution in humans is still insufficient. Fucoidan can either be administered by oral ingestion or by systemic delivery (by intraperitoneal injection, intravenously, or subcutaneously). Although there is much literature available about the biological effects of fucoidan after its administration in the body, barely any research information is available regarding the uptake and fate of fucoidan. Fucoidan is a highly branched molecule and hence difficult to orally absorb. Due to its high molecular weight, there is poor permeation of fucoidan across the human colon adenocarcinoma Caco-2 cell monolayer [15]. Hence, it can be concluded that the distribution of fucoidan in body fluids and tissues may be influenced by molecular weight, branching, and sulphate group position, as well as by the monosaccharide residues and their arrangement. Systematized, biocompatible, biodegradable, in addition to, injectable drug delivery systems are needed for evolution to achieve therapeutic focus in the diseased person, especially on site for a prolonged period of time [16-18]. Collagen, the principal structural protein of the extracellular matrix, may be formed into various forms of delivery systems. The usage of collagen in biomedical applications subsequently progressed rapidly and extended broadly to bioengineering areas due to its excellent biocompatibility and safety [16]. Since the patho-physiology of UC is the ulceration of mucosal and sub-mucosal areas of infected people caused by the extreme deprivation of extracellular matrices, normally, collagen, a major component of spoiled mucosa is regulated by a matrix cascade of metallo proteinsae (MMPs) [19]. Therefore, it is of interest to document data on the synergistic in situ thermosensitive hydrogel formulation for the potential treatment of ulcerative colitis assisted through Sol-gel transition phenomenon.

## Methods and Materials:

### Methods

The Pepsin treated collagen of type I was kindly gifted by the Centre for Human & Organizational Resources Development (CHORD) division, Centre for Academic and Research Excellence (CARE), CSIR-Central Leather Research Institute, Adyar, Chennai. Fucoidan was isolated from the brown seaweed, Turbinaria ornata collected from the coast of the Gulf of Mannar (Rameswaram region, Tamil Nadu, India). The reference standard for in vivo studies, mesalamine, was a kind gift from Glenmark Pharmaceuticals Ltd, Mumbai, India. Dextran Sulphate Sodium (DSS) salt of molecular weight 36-45 kDa was purchased from MP Biomedicals India Pvt. Ltd. In all experiments, aqueous solutions were prepared using deionised water and all chemicals used were of reagent grade.

### Preparation of Type – 1 collagen based fucoidan in situ hydrogel formulate:

Turbinaria ornata, a seaweed biomass, was washed thoroughly with distilled water, shade dried, and pulverized. The extraction and purification were carried out as described elsewhere using a modified method [21]. The carbohydrate containing fractions of sulphated polysaccharides were pooled and analysed for carbohydrate content using fucose as a standard. This crude fucoidan was further purified using a Q-Sepharose fast flow column (4x25 cm) and the eluted solution was dialyzed with water and lyophilized in vials and stored at 4°C.

### Dose selection, formulation and drug administration:

A 10-fold lower dose of fucoidan (200 mg/kg) than the maximum tolerated dose was selected so as to avoid any related safety concerns. A 5 mg/mL solution of collagen was prepared in 20 mM acetic acid and 120 mg of fucoidan was added to this solution with stirring. 50 µL of this solution was administered once daily, intrarectally to each animal in the collagen-fucoidan hydrogel treated group so as to deliver a dose of 8.33 mg/kg of collagen and 200 mg/kg bodyweight of fucoidan, respectively. Likewise the mice in collagen treated group received 50 µL of 5 mg/mL collagen solution in 20 mM acetic acid so as to deliver a dose of 8.33 mg/kg bodyweight of collagen. The fucoidan alone treatment group received 50 µL of 120 mg/mL of fucoidan in distilled water so as to deliver a dose of 200 mg/kg body weight. The vehicle treated group received 50 µL of 20 mM acetic acid solution intrarectally. The stirring temperature maintained at < 10°C and pH was adjusted to 7.2 with 2M Sodium hydroxide using vortex mixer until uniform white suspension formed. The standard drug mesalamine was also formulated as gel using 0.3% carbopol 934 so as to deliver a dose of 20 mg/kg.

### Characterisation of collagen - fucoidan in situ gelling system Tube inversion tests:

2 ml of the collagen-fucoidan hydrogel formulation was transferred into 5 ml sample tube, buffered to pH 7.4 using 1X phosphate buffer saline (PBS) and incubated at 37°C for 2 minutes. The sol-gel state bearing was monitored by inverting the tube post incubation.

### Rheological Study:

This study was carried out to monitor the consistency of the collagen hydrogel after adding fucoidan (120 mg/mL). Generally, Sol-gel transition in the site specific active gel is associated with substantial change in storage (G') and loss modulus (G"). The results were recorded using Anton Paar Physica MCR 301 stress controlled rheometer with the frequency range of 0.1 to 500 rad/s. The temperature of the preheated plate was maintained at 37°C.

### FT-IR characterization of collagen, fucoidan and fucoidan loaded collagen in situ gel:

FT-IR spectra were recorded for collagen, fucoidan and collagen-fucoidan hydrogel combination to monitor any changes in consistency.

### In-vitro release study of fucoidan from collagen – fucoidan hydrogel formulation:

In vitro release of fucoidan from the prepared hydrogel formula at the site of application was estimated by the dialysis method in the simulated physiological environment of the small intestine with a pH of 7.2 using 4% mice caecal buffer solution [25, 26]. Fucoidan release from the collagen-fucoidan hydrogel was monitored using the basket method as described in the United States Pharmacopoeia. Briefly, 2 ml of collagen-fucoidan hydrogel was filled into a dialysis tubing (MWCO 12 kDa, 16mm diameter, Spectrum laboratories, India), with one sealed end. After filling, the other end of the dialysis tubing was sealed, and the dialysis tubing was dipped into the basket and spun at a rotating speed of 100 rpm at 37°C. Three replicates of each formulation were subjected to an in vitro drug release test while maintaining sink conditions. At the time intervals of 0.5, 1, 2, 4, 6, 9, 12, 18, and 24 h, 1 ml of the medium were taken. After each sampling, the same volume of fresh medium was replaced. The solution was filtered through a 0.22 filter membrane, and the amount of fucoidan released was measured using UV spectrophotometry at a maximum wavelength of 560 nm [27]. Assorted kinetic models like zero order, first order, Higuchi model, Korsmeyer Peppas models were employed to monitor drug release kinetics from the developed formulation. Based on the highest regression values for correlation coefficients of the formulations, the best-fit model was determined.

### Stability study of fucoidan loaded collagen hydrogel formulation:

Stability of the prepared formulation was observed for 28 days at 4°C to specify change in color, pH and sedimentation (if any) upon storage. Initially formulation was in milky white color and it was maintained throughout the study. However, slight change in pH and mild sedimentation was observed upon increase in storage time.

### In silico studies:

Docking studies of isolated fucoidan was done using Glide module by the procedure described (Maestro V., 2015) against inflammatory targets like Phospholipase A2, Cyclooxygenase -1 (COX-1) and Cyclooxygenase -1 COX-2, and Lipoxygenase (LOX) as a supporting data or to judge the pathway mechanism in silico. Protein Data Bank (PDB) ID of these targets necessary to perform docking studies was obtained from Protein Data Bank viz., Phospholipase A2 (PDB: 1FV0), LOX (PBD: 3V99), 3N8X and 5KIR for COX-1 and COX-2, respectively.

### Molecular docking studies of fucoidan against Phospholipase A2 (PDB: 1FV0), LOX (PBD: 3V99), COX-1 (PDB: 3N8X) and COX-2 (PDB: 5KIR):

The protein/target LOX (PBD: 3V99) structure co-crystallised with nor dihydroguaiaretic acid (NDGA) was chosen for the docking experiments, which was pre-processed in Maestro software using the Protein Preparation Wizard option. At each stage, the protein structure integrity was checked, missing residues were added using Prime, hydrogen atoms were added to the ligand molecule and bond orders were assigned, hydrogen atoms were added to protein heavy atoms, and so on [28]. The cocrystal ligand was kept during the molecular docking process. The complex was minimised using an optimised potential for liquid simulations 2005 force field until the Root Mean Square Deviation (RMSD) reached 0.3Å. Fucoidan's two-dimensional structure was obtained from the PUBCHEM Database. Using the Ligprep module, it was minimised and geometically refined. Conformers were generated using the torsional search method with distance dependent dielectric solvation treatment and the optimisation potential of the 2005 force field for liquid simulations. Extra precision (XP) docking mode was selected in the Glide application of the Schrodinger software suite for the best fit within the active site of the target. Similarly, Phospholipase A2 (PDB: 1FV0) co-crystallised with aristolochic acid, COX-1 (PDB: 3N8X) co-crystallised with nimesulide, and COX-2 (PDB: 5KIR) co-crystallised with rofecoxib were chosen and pre-processed with various steps to prepare it for docking experiments, as previously mentioned. The extra precision (XP) docking mode was also used in this case, and the results were tabulated.

### In vivo efficacy testing of collagen-fucoidan in situ gelling system by DSS induced colitis method in mice model

The in vivo efficacy testing of the collagen-fucoidan hydrogel was performed using DSS induced colitis in mice according to the institutional animal ethics committee (IAEC/49/SRU/513/2016). All the animals (n = 8) were divided into 6 groups. The mice in the vehicle and other treatment groups were exposed to 4% DSS solution in autoclaved drinking water ad libitum, while the healthy control group received autoclaved drinking water for the entire length of the study (7 days). The animals were grouped as mentioned in Table 1(see PDF). Group 1 served as the healthy control group, group 2 served as the vehicle control group, group 3 was treated with fucoidan at 200 mg/kg body weight, group 4 mice received collagen (5 mg/ml in 20 mM acetic acid), group 5 mice received the collagen-fucoidan hydrogel (containing 120 mg/mL fucoidan in 5 mg/ml collagen in 20 mM acetic acid solution), and group 6 mice received mesalamine suspension (in 0.3% carbopol 934) dosed at 20 mg/kg bodyweight. The collagen, fucoidan, collagen-fucoidan hydrogel, and the reference mesalamine suspension were administered by rectal route once daily from day 1 of the 4% DSS exposure for the entire length of the study (7 days). The DSS solution was prepared using autoclaved water (pH 8.5) and sterile filtered drinking water. The DSS solution in drinking bottles was discarded every 2-3 days and a fresh 4% DSS solution was supplied.

Animals were monitored daily for body weight, stool consistency, the presence of blood in the stools and colitis severity scoring. At the end of the study, mice in all groups were sacrificed, and necropsy was carried out to collect the colons from all mice. The colons were washed to remove the faecal matter with 1X PBS, and the colon lengths were measured using a scale. The colon tissue collected from all mice was fixed in a 10% buffered formalin solution prior to processing for histopathological analysis by microscopy.

### Clinical Score:

A clinical colitis scoring procedure was adopted, as reported elsewhere, [29] and severity scoring on a scale of 0-3 was performed. Normal stool consistency with negative hemoccult was scored as: 0; soft stools with positive hemoccult: 1; very soft stools with traces of blood: 2, and watery stools with visible rectal bleeding was given a score of: 3.

### Histological Score:

At study termination, mice in all groups were sacrificed using CO2 asphyxiation followed by necropsy and the colon was collected and fixed for 7 days in 4 % paraformaldehyde (PFA). The tissues were processed and embedded in paraffin, transverse sections (3-5 µm) mounted on microscopy slides and stained with hematoxylin and eosin and histopathological evaluation and scoring was carried out using microscopy. All tissue samples mounted on the slides were examined for mucosal surface epithelium cryptitis and crypt branching. The following scoring system for determining disease severity was used for histopathological examination in the study. 1- Minimal; 2- Mild; 3- Moderate; 4- Marked; 5-Severe;

### Statistical Analysis:

Statistical analysis of data was done by Graph Pad software using one way ANOVA with Tukey multiple comparison. All groups were compared with each other for significance.

## Results and Discussion:

Upon isolation from selected seaweed Turbinaria ornate, the percentage yield of fucoidan was 3.29 ± 0.19%. The carbohydrate content of the fucoidan after isolation and purification was 59.76±0.27% (F1 to F10, gradient decrease in the polysaccharide amount).

## Characterisation of fucoidan collagen in situ gel Tube Inversion test

The sol-gel transformation activity was recorded 2 min after the addition of fucoidan to the collagen solution in 20 mM acetic acid. Upon tube inversion testing, a gelling effect was observed at physiological pH of 7.4 (Figure 1 - see PDF). This gelling effect may be attributed to the collagen fibril structure at physiological pH and could be expected to retain the gel consistency at the rectal pH of 7-8 and temperature of 37°C. A sol-gel reversibility phenomenon was observed when the temperature was dropped to 4°C, where the gel form was converted to fluid form. These findings allowed us to confirm that the formulation could continue to remain in the gel state at physiological temperatures in vivo.

## Rheological study

Sol-gel-sol transformation of in situ gel follows a major transition shift of modulus for storage/loss (Figure 2 - see PDF). The strain-stress curves are expressed as tan δ that represents the ratio of G"/G' or storage to loss modulus of the formulated gel, respectively [30, 31]. Accordingly, the formulations at pH 4 and pH 7 yielded tan δ value of 0.62 and 1.23, respectively. The storage modulus values were higher than 1 at pH 7.4 indicating that the formulation would retain hydrogel consistency in vivo. This hydrogel consistency may be attributed to ionic and non-covalent bond interaction between collagen and fucoidan [32, 33]. Thus, non-Newtonian linear viscoelastic properties of the hydrogel formulation may contribute to the obtained tan δ values suggesting that both collagen and collagen fucoidan combination may yield a attain hydrogel consistency at physiological pH.

## FT-IR characterization:

The FTIR spectrum of fucoidan, collagen, and collagen-fucoidan in situ hydrogel are shown in Figure 3a, 3b, and 3c(see PDF), respectively. Spectra of fucoidan Figure 3(a)(see PDF) shows characteristic bands at 836 cm-1 corresponding to C-O-S bending vibration and sulphate substituent at axial C-4, 2930 cm-1 to C-H stretching (C-6 group of fucose, galactose and pyranose ring), 1051 cm-1 indicates C-O stretching vibrations of Glycosidic bonds, 1259 cm-1 to C-C stretching vibrations of the pyramid ring and 3454 cm-1 corresponds to H-H bonded-H stretching vibrations. Figure 3(b)(see PDF) indicates the IR spectrum of collagen, which shows a characteristic band at 1450 cm-1 of C-H bending modes, 3400 cm-1 for N-H stretching (Amide A band), 1540 cm-1 of Amide II (N-H bending), and 1655 cm-1 and 1248 cm-1 for Amide I (C=O stretching) and Amide III (C-N stretching) respectively. Hence, in freeze dried formulated hydrogel Figure 3(c)(see PDF), all major peaks corresponding to both fucoidan and collagen are retained without any shift in the region, indicating no interaction between fucoidan and collagen. Figure 3(d)(see PDF) shows the overlaid FTIR Spectra of Collagen, Fucoidan, and Collagen-Fucoidan Hydro gel.

## In-vitro release study of fucoidan from collagen – fucoidan hydrogel formula

The Fucoidan release profile from the formulated hydrogel was shown in Figure. 4a and 4b.(see PDF) In about 24 hours, 98.99% and 6 hours, 99.05% of the drug was released from the gel and suspension formulations, respectively. The dissolution time (DT), DT 50, and DT 80 for the gel were found to be 5 hours and 15 hours, respectively. Drug release was significantly delayed in the in situ gel system (with collagen) when compared to the suspension form. Thus, the sol to gel transition phase may have led to delayed drug release during dissolutions. The release from the gel is significantly prolonged when compared to the suspension form. In addition, the in vitro release mechanism of fucoidan from this gel was further evaluated. The results show that the fucoidan release from in situ gel follows the Higuchi square root law, i.e., the amount of drug released is proportional to the square root of the time [34]. A linear correlation (r2 = 0.98) observed indicates that the drug release from the collagen-fucoidan hydrogel delivery system followed a matrix diffusion controlled mechanism. Thus, the diffusivity of the drug is constant, and perfect sink conditions could be expected to be attained in the release environment.

## Stability study of hydrogel formulation:

For stability studies, the collagen-fucoidan hydrogel formulation was sampled on day 0, 3, 7, 14, 21, and day 28. The gel remained milky white in colour throughout the period of refrigeration as indicated in Table 2(see PDF). However, minor changes in pH as well as slight turbidity were detected upon increasing storage time for periods > 28 days. The collagen-fucoidan hydrogel was stable (at 98% or more) for a period of 28 days. Adequate care was taken to avoid exposure of the prepared hydrogel to temperatures above 10°C to prevent collagen fibril formation or avoid loss of consistency of the formulation.

## In silico studies:

In silico docking investigations were done utilising Glide application of Schrodinger software package for the pure fucoidan against selected inflammatory targets such Phospholipase A2 (PDB: 1FV0), COX-1 (PDB: 3N8X), COX-2 (PDB: 5KIR) and LOX (PBD: 3V99) (PBD: 3V99).

Table 3(see PDF) illustrates the value of docking score and glide energy of fucoidan and mesalamine with all four selected targets. Comparing the docking score of all the targets, fucoidan reveals high docking score in COX-1 with -7.67 kcal/mol and glide energy of -38.86 kcal/mol. Figure 6(see PDF) projects the interactions of fucoidan and mesalamine against the active site of COX-1 (PDB: 3N8X) where it has hydrogen bonds with ARG120 (2.95Å) and TYR355(2.79Å) residues. Also Figure 5, Figure 7 and Figure 8 (see PDF) project the interaction with PLA-2, LOX and COX-2. Hence in silico molecular docking study results against the specified targets provides as a supporting data to anticipate the potential pathway mechanism. Accordingly, COX-1 has strong docking score and hydrogen bond distance with fucoidan. Hence fucoidan displays its anti-inflammatory effectiveness by suppression of Cyclooxygenase-1 (COX-1) activities.

## In vivo efficacy of in situ fucoidan loaded collagen hydrogel:

Based on the literature of acute toxicity studies carried out as per the OECD guidelines 420, oral dose toxicity was limited to 2000 mg/kg body weight [22]. Fucoidan has been reported to be non-toxic when given at doses of up to 2000 mg/kg bodyweight [24]. Also, fucoidan from Udaria species was approved by the USFDA and recognised as a safe category food ingredient with a limit of up to 250 mg/day [23]. The collagen dose of 8.33 mg/kg was chosen based on a previous study on DSS-induced colitis in mice.

The site-specific synergistic anti-inflammatory potential of fucoidan and collagen was monitored using DSS induced colitis in mice. 44 female BALB/c mice were assigned to six groups. The following results were obtained under the experimental conditions: Mortality was not observed in animals from different groups during the study. All the animals treated at the intended dose levels were observed to be normal throughout the experimental period. In the DRF study, on day 0, there was no significant reduction in body weight in all groups. From day 4th to 7th, there was a significant decrease in body weight in all animals at 4% DSS. In the efficacy testing study of the formulation, on day 0, there was no significant reduction in body weight in all groups. On day 4, there was a significant decrease in body weight in all groups except the control group. Disease control showed a significant decrease in body weight in all animals. The test drug groups for Fucoidan, collagen, and formulation were found to be statistically significant in terms of increase in body weight from day 5 to day 11. Figure 9a and 9b(see PDF) represent the body weight of animals in the dose finding study (DRF) and the body weight of animals in the efficacy study. Hence, based on the study results, there was a reduction in body weight after colitis induction in all groups, and recovery or weight regain was observed in various treatment groups based on the efficacy of the compounds. In particular, on day 11, there was complete recovery in the G5 as compared to negative control. All groups were compared with each other for significance. Likewise, clinical signs were recorded once a day, daily. Table 4 shows the clinical activity grading scheme for rectal bleeding and stool consistency. Rectal bleeding and blood clots around the anus were noted by eye inspection till experimental completion. Figure 10(see PDF) represents the clinical signs and colitis score in the DRF study and efficacy study, respectively. Clinical signs and colitis score for stool consistency significantly decreased on day 11 for the treatment group (G5) compared to control group negative control (G1). There was also a considerable drop in rectal bleeding in the test group during the treatment when compared to the reference group (G2) (p < 0.01) as well as with animals that received fucoidan (G3) and collagen (G4) alone. This could be due to the synergistic effect of fucoidan and collagen on site, creating profuse protein in the colonic mucosa and thereby assisting its faster regeneration.

## Necropsy:

At the end of the experimental period, mice from each group were euthanized and subjected to necropsy, and the colon was collected. Histopathology was also carried out. Pictures of DSS-induced colitis from mice of different groups after necropsy are shown in Figure 11(see PDF).

Histological section image of all groups (G1-G6) was shown in Figure 12a - 12f(see PDF). The following grading system was used for histopathological examination in the study. Individual Animal histopathology findings and summary of incidence and severity of histopathology are annexed as Minimal, Mild, Moderate, Marked and Severe. Histological analysis of distal colon section of mice from normal control group (G1) has normal histology of mucosal, submucosal and muscularis mucosal layer, crypts and goblet cell with nil mucosal damage and no inflammatory sign compared to DSS treated disease control group (G2) with widespread mucosal damage and marked severity of inflammatory cell infiltration in submucosa and loss of goblet cells, shortening and distortion of crypts. Similarly distal colon region of drug treated group animals were assessed after isolation. Fucoidan treatment group (G3) revealed moderate severity of submucosal inflammatory cell infiltration, goblet cells loss, shortening and distortion of crypts when compared with disease control animals. Collagen treatment group (G4) displayed mild severity of mucosal and submucosal inflammatory cell infiltration) with normalization of villi, crypts and goblet cells, Fucoidan + Collagen treatment Group (G5) exhibited normalization of mucosa, submucosa, muscularis mucosa, crypts and goblet cell. Finally, standard Mesalamine Treatment Group (G6) showed normal histology when compared with disease control animals.

## Conclusions:

The concept of sol-gel transition of collagen hydrogel for biomedical application phenomenon has already been developed. Based on the literature, acid soluble collagen solution following alteration in their pH and temperature converted into a transparent gel. Hence in situ fucoidan loaded collagen hydrogel was prepared and it was investigated for its efficacy for the treatment of UC. Prepared in situ gel exhibited sustained release for roughly 12 hours with a considerable reduction in mucosal damage as well as clinical score for rectal bleeding when compared with the standard. As collagen, the principal component of damaged mucosa in the state of Ulcerative colitis deteriorated the combined therapy of fucoidan and collagen may function synergistically and might provide an efficient solution on site.

## References

[1] Lean QY (2015). PLoS One..

[2] Travis SPL (2008). J Crohns Colitis..

[3] Meier J, Sturm A (2011). World J Gastoenterol..

[4] Ardizzone S (2010). Therap Adv Gastroenterol..

[5] Conrad K (2014). Autoimmun Rev..

[6] Loft S, Poulsen HE (1999). Methods Enzymol..

[7] Sturniolo GC (1998). Scand J Gastroenterol..

[8] M'koma AE (2013). Clin Med Insights Gastroenterol..

[9] Na SY, Moon W (2019). Gut Liver..

[10] Ahmad T (2021). Mar Drugs..

[11] Li B (2008). Molecules..

[12] Rocha de Souza MC (2007). J Appl Phycol..

[13] Meenakshi B (2013). Phtomedicine.

[14] Shaibi KMM (2013). Appl Biochem Biotechnol..

[15] Boo HJ (2011). Phytother Res..

[16] Natarajan V (2012). Int J Bio Macromol..

[17] Lee CH (2001). Int J Pharm..

[18] Meyer M (2019). Biomed Eng Online..

[19] Wang YD, Mao JW (2007). World J Gastroenterol..

[20] Wang YD, Yan PY (2006). World J Gastroenterol..

[21] Duarte ME (2001). Carbohydrate Res..

[22] Kim KJ (2010). Toxicology..

[23] Citkowska A (2019). Mar Drugs..

[24] Jung YM (2008). Toxicol Res..

[25] Kotla NG (2016). Int J Nanomedicine..

[26] Sinha VR (2004). Int. J. Pharm..

[27] Hongying LIU (2002). Journal of Ocean University of Qingdao..

[28] Elokely KM, Doerksen RJ (2013). J Chem Inf Model..

[29] Walmsley RS (1998). Gut..

[30] Ikeda J (2021). Gels..

[31] Kim EJ (2016). Bio macromolecules..

[32] Berger J (2004). Eur J Pharm Biopharm..

[33] Nadege B (2005). Biomacromolecules..

[34] Higuchi WI (1962). J Pharm Sci..

